# Heavy Metal Distribution in Street Dust from Traditional Markets and the Human Health Implications

**DOI:** 10.3390/ijerph13080820

**Published:** 2016-08-13

**Authors:** Jin Ah Kim, Jin Hee Park, Won Ju Hwang

**Affiliations:** 1 College of Nursing Science, Kyung Hee University, 26, Kyungheedae-ro, Dongdaemun-gu, Seoul 02447, Korea; kimja@khu.ac.kr; 2 Geologic Environment Division, Korea Institute of Geoscience and Mineral Resources, 124, Gwahak-ro, Yuseong-gu, Daejeon 34132, Korea; 3 East-West Nursing Research Institute, College of Nursing Science, Kyung Hee University, 26, Kyungheedae-ro, Dongdaemun-gu, Seoul 02447, Korea; hwangwj@khu.ac.kr

**Keywords:** heavy metal, marketplace, street, dust, health, depression

## Abstract

Street dust is a hazard for workers in traditional markets. Exposure time is longer than for other people, making them vulnerable to heavy metals in street dust. This study investigated heavy metal concentrations in street dust samples collected from different types of markets. It compared the results with heavy metal concentrations in heavy traffic and rural areas. Street dust was significantly enriched with most heavy metals in a heavy traffic area while street dust from a fish market was contaminated with cupper (Cu), lead (Pb) and zinc (Zn). Street dust from medicinal herb and fruit markets, and rural areas were not contaminated. Principal component and cluster analyses indicated heavy metals in heavy traffic road and fish market dust had different sources. Relatively high heavy metal concentration in street dust from the fish market may negatively affect worker’s mental health, as depression levels were higher compared with workers in other markets. Therefore, intensive investigation of the relationship between heavy metal concentrations in street dust and worker’s health in traditional marketplaces should be conducted to elucidate the effect of heavy metals on psychological health in humans.

## 1. Introduction

Heavy metal containing dust is a health issue in both industrial and urban area. Motor vehicles and building smoke are the main sources of heavy metals in urban dust [1]. Heavy metal distribution in urban dust in many countries, including China [2,3], Hong Kong [4], India [5], Korea [6,7], Spain [8], Jordan [9] and Mexico [10] has been investigated. In China, urban soils and road dust are contaminated with chromium (Cr), nickel (Ni), Cu, Pb, Zn and cadmium (Cd). Traffic and industrial emissions are the main sources of heavy metal pollution [11]. Dust from China is transported to surrounding countries, including Korea, and this polluted dust seriously affects human health [12]. East Asia has experienced a serious dust problem each spring over the past decade, with dust being transported long distances. 

Even though there have been extensive studies on heavy metal contamination of dust in urban areas, no research has been performed on the heavy metal distribution of dust in traditional marketplaces and the implications for human health. Heavy metal distribution may vary between the different types of traditional markets because the products sold in each carries different levels of heavy metals. Some medicinal plants are contaminated with heavy metals such as Cd and Pb [13]. Chinese herbal medicines can contain potentially toxic levels of arsenic (As) and Cd [14]. Not only medicinal plants, but also vegetables and fruit can be contaminated with heavy metals depending on corresponding soils [15]. Some fresh and marine fish species purchased from markets in Hong Kong had elevated concentrations of Cd, Pb and mercury (Hg) [16]. Therefore, street dust from different traditional markets could be enriched with different heavy metals of varying concentrations and affect the health of humans working in the market.

Market workers are constantly exposed to street dust, and the heavy metals in the dust may affect their health. This is particularly so in traditional marketplaces where people work outside and are exposed to the dust throughout their working hours. A Korean survey of five hundred workers at traditional markets reported average working hours to be 11.1 h/day, longer than statutory working hours; the mean work experience was 19.0 years [17]. Moreover, this survey indicated the percentage of street vendors was almost 35% [17]. Theses mean that market workers are vulnerable to heavy metal containing dust exposure. 

Human can be exposed to heavy metals through dermal adsorption and the ingestion of dust particles [18]. Heavy metals may cause DNA damage, thereby resulting in mutagenic, teratogenic and carcinogenic effects in human health. Cadmium, As, and Cr can be carcinogenic and Pb affects the nervous system and brain [19]. Symptoms of long-term Pb exposure are memory deterioration, prolonged reaction time and reduced intellectual capacity [20]. Furthermore, the systematic review on effect of long term outdoor air pollution to psychological functions shows that ambient air pollution is associated with mood disorders, neurocognitive function and neurodegenerative disease in long term exposed people [21]. Therefore, considering the work conditions of workers at traditional marketplaces, not only their physical health but also their mental health could be threatened because of the polluted dust. Nevertheless, studies on mental health of traditional market workers in relation to heavy metals in street dust are limited. 

The hypothesis of this study was that heavy metal concentrations in street dust vary depending on the type of market, and that market workers have heavy metal-related mental health problems. The objective was to investigate the level of heavy metals in the dust of traditional marketplaces and to compare their levels with those of heavy traffic and rural areas. In addition, the heavy metal concentrations were related to mental health survey results of traditional market workers.

## 2. Methods 

### 2.1. Dust Sampling

**Figure 1:**
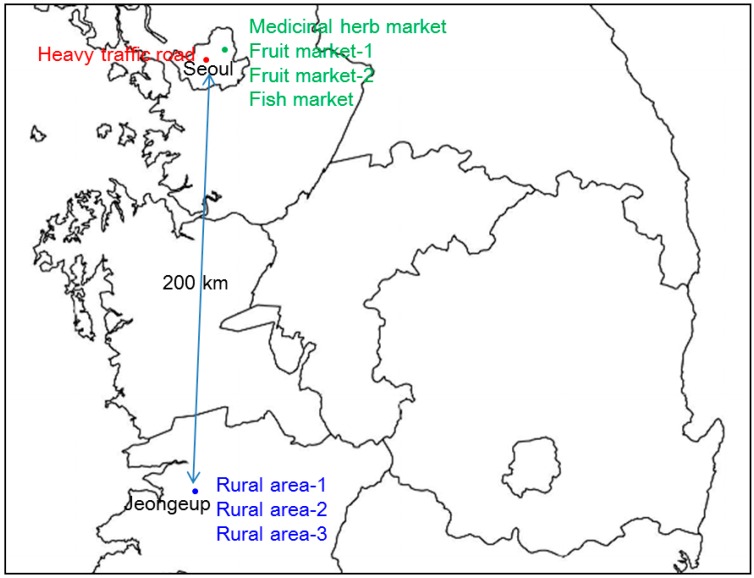
Sampling locations: a medicinal herb market, a fish market, two fruit markets, and a heavy traffic area in Seoul, and rural areas in Jeongeup, South Korea.

Three different sites (food marketplaces and a heavy traffic area in Seoul and a rural area in Jeongeup, south area of South Korea) were selected for dust sampling. Samples were collected during winter season at −6.6 °C and 45.5% relative humidity (December 2014). Eight samples were collected depending on traffic volumes, urbanization and type of markets. Four different food markets, including two fruit markets, one medicinal herb market, and one fish market, were selected to evaluate the relationship between heavy metals in dust and human health problems. Both heavy traffic and rural roads were selected for comparison with samples from traditional markets. Sampling locations are marked in Figure 1. Deposited dust was collected with a plastic brush and tray from 3 to 5 points at each sampling site and then composited. The dust samples were air-dried, and sieved to less than 150 µm to remove large soil particles. 

### 2.2. Analysis of Dust Samples

Dust samples (0.2 g) were ground and weighed into a Teflon vessel. Concentrated HCl (3 mL), HNO_3_ (2 mL), HClO_4_ (1 mL) and HF (2 mL) were slowly added to the Teflon vessel. Teflon vessels were placed on an aluminum heating block preset to 110 °C and heated to incipient dryness. The temperature was raised to 160 °C and samples heated to complete dryness. Teflon vessels were removed from the heating block and allowed to cool. After cooling 1 mL of concentrated HNO_3_ and 19 mL of 1% HNO_3_ were sequentially added and the vessels capped and heated in a drying oven preset at 100 °C for 30 min. Elemental concentrations in the digested solution were analyzed using Inductively coupled plasma—optical emission spectroscopy (ICP-OES; Perkin Elmer 5300DV, Waltham, MA, USA). To minimize error, blank samples were analyzed simultaneously with tested samples. 

### 2.3. Statistical Analysis of Heavy Metal Concentrations

The enrichment factor (EF) of each heavy metal in the street dust samples was calculated based on the standardization of a measured heavy metal against a reference element. A reference element is a conservative element; commonly used reference elements include aluminum (Al), silicon (Si), iron (Fe), manganese (Mn), scandium (Sc), titanium (Ti), etc. [22,23]. In this study, Fe was used as a reference element and reference elemental concentrations were taken from the chemical composition of the continental crust [24]. The EF of a heavy metal in a street dust sample can be defined as in Equation (1).

(1) $$\mathrm{EF}=\frac{\left[\frac{C_{e}}{C_{ref}}\right]Sample}{\left[\frac{C_{e}}{C_{ref}}\right]Background}$$

where *C_e_* is the concentration of the element of interest and *C_ref_* is the concentration of reference element [2]. 

All experiments were carried out in duplicate and the mean of duplicate analysis used for statistical analysis. Data were evaluated using principal component analysis (PCA) and cluster analysis (CA) with PASW 18.8 software. Principal component analysis is widely used to reduce the number of variables and to extract a small number of components to represent the observed variables. Cluster analysis is used to classify samples based on distances and similarity among samples. These statistical techniques were used to evaluate the latent relationships between variables and samples in street dust [25]. The PCA with varimax rotation and Kaiser Normalization were applied to the data matrix and the hierarchical CA was conducted with Euclidean distance. 

### 2.4. Questionnaire Investigation

Individual interviews were conducted by three trained researchers for 163 workers from three traditional marketplaces in Seoul, South Korea; 38 from a fish market, 73 from the fruit markets and 52 from a medicinal herb market. After the study’s purpose, confidentiality, and the fact that the data would be used only for the stated purposes, signed agreements were collected from tradespeople who understood the study’s purpose and volunteered participation. Data were collected using a structured questionnaire that consisted of general characteristics such as age, work related factors including job type and working hour, and depression symptoms. Depression level was measured using the Korean version of the Center for Epidemiologic Studies Depression (CES-D) Scale, developed by Radloff [26] and translated by Cho and Kim [27]. The total score range was 0–60; a higher score relates to a higher degree of depression. Data were collected after the Institutional Review Board at K University granted permission (IRB No. KHSIRB-13-035).

### 2.5. Statistical Analysis for Questionnaire Investigation

Statistical analysis was performed using SPSS 22.0 (SPSS Inc., an IBM Company, Seoul, Korea). Data were summarized using the mean and standard deviation for continuous variables. Comparisons among the three different markets for variables were performed using an ANOVA. 

## 3. Results and Discussion

### 3.1. Total Elemental Concentrations

Street dust samples were analyzed for Al, sodium (Na), magnesium (Mg), potassium (K), calcium (Ca), Mn, lithium (Li), Cr, Fe, Co, Ni, Cu, Zn, As, Cd, Pb and molybdenum (Mo) to evaluate metal contamination of the dusts from different sampling sites. Among these elements Li, Co, As, Cd and Mo concentrations were less than the quantification limit (20 mg/kg) in dust samples and were not included in the results and statistical analysis. Aluminum and K concentrations were low in samples from the heavy traffic area while Na, Mg and Ca concentrations were high in these samples (Table 1). According to the background elemental concentrations of uncontaminated surface soils in USA, all elements were within the maximum concentration [28]. However, the Na concentration in the heavy traffic area was close to the maximum value, K concentrations in rural areas were relatively high, as were Ca concentrations in the heavy traffic road and fish market and Fe in fish market dust. 

Cations such as Na, Mg and Ca in the heavy traffic area sample can be related to traffic, coal combustion, road pavement materials and the use of de-icing materials [29,30,31,32]. Heavy metals such as Ni, Cu, Zn, and Pb in the street dust of the heavy traffic area were much higher in concentration than those of rural areas. Ni and Cu concentrations in the street of the heavy traffic area exceeded anxiety criterion and Zn concentration exceeded the action level of soil pollution measures of Korea [28]. Previous study also reported concentrations of Cu, Zn and Pb in roadside soils were significantly higher than these in rural areas [30]. Heavy metal concentrations in street dust from fruit markets had similar values to the rural areas. Nickel and Cu concentrations in the street dust of the medicinal herb market were slightly higher than those of rural areas. Street dust from the fish market had the highest Zn and Pb concentrations, twice that of the heavy traffic area. Zinc concentrations of the heavy traffic road and fish market exceeded the action level (900 mg/kg) of soil pollution measures of Korea [33]. Copper and Pb concentrations in the fish market dust exceeded the anxiety criterion (Cu 150 mg/kg, Pb 200 mg/kg) [33]. 

Heavy metal concentrations were higher in the fish market than in other markets and can be attributed to the deposition of leachate from contaminated fish. The study on metal concentration of marine fish in China reported that Pb and Cd concentrations in some fish species purchased from markets exceeded the international standard concentrations of the European Union and the China National Standard Management Department [16]. Zinc and Cu concentrations in fish species in China ranged from 15.7 to 29.5 mg/kg and from 0.79 to 2.26 mg/kg, respectively. Although the concentrations were within the permissible limits for human consumption according to the international guidelines [34], continuous deposition of fish leachate may have led to contamination of the street dust. Also, Chinese studies [35,36] reported the concentration of Zn was the highest among heavy metals in fish in Taihu Lake and Tianjin, China. The Zn concentration in fish from Taihu Lake was reported as higher than the Chinese Food Health Criterion [35]. Korea imports 40.0% of fish in market from China [37]. This explains how concentrations of heavy metals such as Pb, Cu, and Zn could increase in fish market dust over other marketplaces.

### 3.2. Enrichment Factors of Heavy Metals

**Figure 2:**
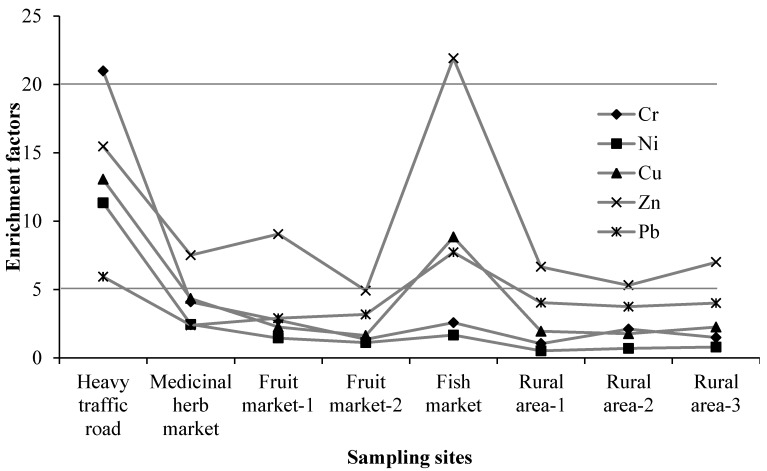
Enrichment factors of heavy metals in street dust samples.

Enrichment factors can be used to differentiate anthropogenic contamination from a natural origin because it is calculated based on reference to elements in the earth’s crust [10]. To find out which elements were enriched due to anthropogenic contamination, EFs were calculated and are presented in Figure 2. Although there is no accepted categorization system for EFs, contamination categories can be grouped based on the EFs: EF < 2 indicating deficiency to minimal enrichment, EF = 2–5 moderate enrichment, EF = 5–20 significant enrichment, EF = 20–40 very high enrichment and EF > 40 extremely high enrichment [2,23]. Street dust from the road with heavy traffic was significantly enriched with most heavy metals. The fish market street dust was significantly contaminated with Zn, indicating a stronger contribution from anthropogenic sources. In most samples EFs of Zn were greater than 5, suggesting significant enrichment of Zn. The EFs of Cu and Pb in fish market dust were also greater than 5 implying significant contribution from anthropogenic sources.

### 3.3. Principal Component and Cluster Analyses

**Figure 3:**
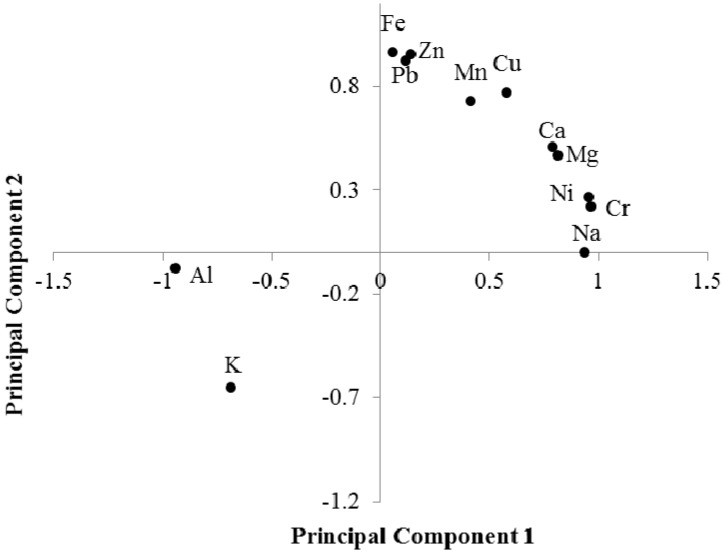
Plot of loadings of the first two principal components in principal component analysis.

Principal component and cluster analyses were conducted to evaluate relationships among elements and samples. A correlation matrix of street dust samples is shown in Table 2. Among heavy metals, Cr and Ni, Cu and Zn, Cu and Pb, Pb, and Zn were significantly, positively correlated, indicating they originated from the same traffic and industrial sources. Significant principal components were extracted from eigenvalues and eigenvectors; two principal components with eigenvalues > 1 explained 89.6% of total variance. Principal component one explained 48.6% of total variance and was strongly correlated with Al, Na, Mg, Ca, Cr, and Ni (Figure 3). Chromium and Ni were highly correlated with major cations in dust samples, indicating these were from natural sources. Principal component 2 accounted for 41.0% of total variance and indicated high loadings for Mn, Fe, Cu, Zn, and Pb. High loadings of Mn and Fe reflected the bulk matrix of the street dust whereas heavy metals such as Cu, Zn and Pb were correlated with matrices from different contamination sources such as traffic and industry (Figure 3) [38]. 

**Figure 4:**
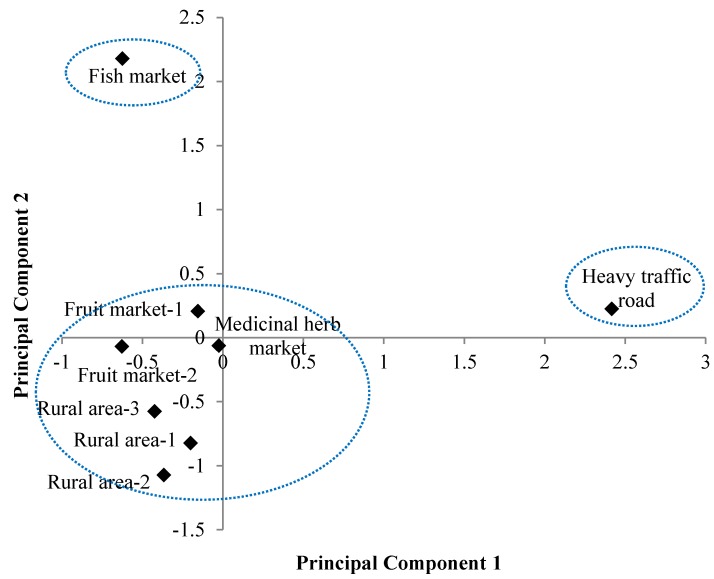
Principal component score plot of the street dust in the projection of principal components 1 and 2.

The principal component score was calculated to provide a geochemical index for hazardous street dust samples. The principal component score plot in Figure 4 describes the characteristics of street dust samples. Samples were grouped in three different clusters. Samples from the fish market and heavy traffic road showed high PC1 and PC2 values suggesting these samples could be hazardous. The results were in good agreement with the EFs.

**Figure 5:**
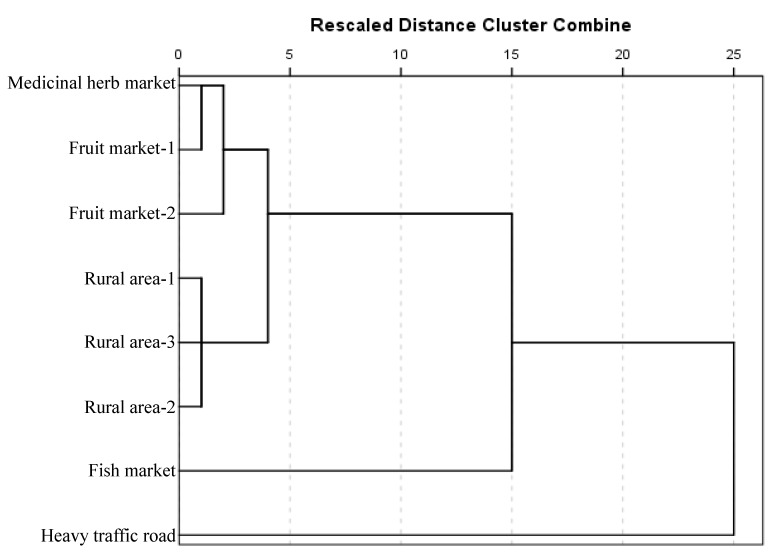
Dendrogram showing the clustering of the street dust samples. Similarities have been calculated from Euclidean distance.

Hierarchical cluster analysis was used to identify relatively similar groups of samples. Figure 5 shows a dendrogram of clusters using Euclidian distance and displays three different clusters. Street dust from the fish market and heavy traffic road were divided into different groups. Street dust samples from medicinal herb and fruit markets, and rural areas, joined together at a relatively higher level. The result yielded three clusters, as for the principal component score plot (Figure 4). The CA also indicated street dust from the heavy traffic road was different from the fish market dust, indicating the sources of contamination differed, as discussed in Section 3.1. 

### 3.4. Relationship between Heavy Metal Concentration in Dust and Human Health

A questionnaire investigation was conducted to evaluate the possible effect of heavy metals in street dust on the mental health of market workers. The mean depression level of workers in the fish marketplaces was 21.1, which was significantly higher than those of medicinal herb (16.9) and fruit (14.3) marketplaces (*p* < 0.001). Various factors can be related to the depression level of workers. Work-related factors such as long working hour [39] and poor work environment, including exposure to dust, noise, and high to extreme temperatures [40] could be risk factors for mental health. Old age may also affect mood disorders, including depression [41]. This study found the average daily hours of workers in traditional marketplaces was 11.4 h and the mean work experience was long as 21.6 years. Also, street vendors made up 34.4% of workers. Moreover, the mean age of workers in this study was 59.4 years (Table 3). Workers at traditional markets are more vulnerable to psychological health disorders, like depression, because of long work hours, poor work conditions and old age. 

Despite the similar age and working conditions of vendors in medicinal herb, fruit and fish markets, the level of depression was significantly higher among workers in the traditional fish market (Table 3). This suggests factors other than working conditions may have affected the levels of depression in fish market workers. Some heavy metals such as As, Cd, Pb, Cu and Zn seriously affect human health. Lead is more harmful to mental health than the other heavy metal. People with high blood Pb levels showed increased odds of major depression and panic disorders [42]. A recent study also reported that blood Pb level was associated with clinically diagnosed mental disorders, especially mood disorders, in an Asian population [43]. Eum et al. [44] associated low-level Pb exposure with increased depression and phobic anxiety among premenopausal women and women who took hormone replacement therapy. The elevated levels of Pb recorded in the fish market dust, in this study, could explain the higher levels of depression in the fish market workers interviewed. However, high metal concentrations cannot solely explain the level of depression. Since depression is a notably non-specific mental problem, the nature of daily work and harsh working environments (i.e., gutting fish, smelly work environment, economic concerns associated with selling a highly perishable product) may also affect the level of depression. Therefore, the influence of other environmental conditions on depression of fish market workers should be considered in future studies.

In addition, zinc concentration was the highest in the street dust of fish market. Long term exposure to high levels of Zn is a risk factor for physical health problems such as lethargy, respiratory disorder, diarrhea, and prostate cancer [45]. Although Ni, Cu and Zn concentrations were also high in the street dust from the heavy traffic area, fish market workers would be continuously exposed to the dust for longer than people walking on a heavy traffic road. Therefore, physical health problems may be present due to elevated Zn concentration in this group, but not evident in the general population making use of contaminated heavy traffic roads. Consequently, further studies on relationship between heavy metal concentration and physical health are recommended. 

## 4. Conclusions 

Street dust in the fish market had elevated levels of heavy metals Cu, Ni, Pb and Zn, more so than other markets. Lead concentrations exceeded the anxiety criteria and Zn concentration exceeded the action levels of soil pollution measures of Korea, which could affect the physical and mental health of those working in fish markets. Depression is one of the health risks of exposure to elevated heavy metals. The mean depression level of workers in the fish market was 1.5 times higher than the workers in other marketplaces. However, as this is a cross-sectional study with a small sample size, the cause-and-effect relationship between heavy metal contamination and health risks cannot be inferred. Metal concentration in blood, hair, or nail samples could not be analyzed in this study because of ethical issues. Further research, such as investigating blood heavy metal concentrations, is needed to determine the effect of heavy metal exposure on human health in this population. Furthermore, detailed information on previous occupational metal exposure, the nature of work and environmental conditions of the work would support the relationship between heavy metals in street dust and depression. Follow-up studies comparing fish sellers who worked as street vendors and those who worked indoors are suggested to elucidate the effect of heavy metals on depression level.

## Figures and Tables

**Table 1:** Total elemental concentrations in street dust samples collected from a heavy traffic road, marketplaces and rural areas.

Samples	Al	Na	Mg	K	Ca	Fe	Mn	Cr	Ni	Cu	Zn	Pb
	(wt %, Mean ± SD)						(mg/kg, Mean ± SD)					
Heavy traffic road	4.13 ± 0.23	6.38 ± 0.47	1.39 ± 0.12	1.88 ± 0.17	6.85 ± 0.49	3.79 ± 0.22	769 ± 2	794 ± 75	245 ± 47	353 ± 3	1188 ± 18	128 ± 1
Medicinal herb market	6.99 ± 0.66	1.44 ± 0.12	0.96 ± 0.08	3.01 ± 0.29	1.99 ± 0.14	3.58 ± 0.30	641 ± 4	146 ± 11	50 ± 3	111 ± 2	544 ± 52	49 ± 2
Fruit market-1	7.20 ± 0.01	1.55 ± 0.12	0.92 ± 0.00	2.77 ± 0.25	2.49 ± 0.64	3.80 ± 0.01	772 ± 27	105 ± 0	31 ± 4	61 ± 2	697 ± 34	63 ± 6
Fruit market-2	9.28 ± 0.02	1.92 ± 0.08	0.71 ± 0.01	3.68 ± 0.11	1.75 ± 0.08	3.73 ± 0.04	581 ± 65	51 ± 4	24 ± 0	44 ± 2	372 ± 25	68 ± 0
Fish market	8.18 ± 0.91	1.87 ± 0.19	0.88 ± 0.10	2.62 ± 0.25	4.75 ± 0.49	5.46 ± 0.63	723 ± 11	140 ± 5	52 ± 3	344 ± 32	2425 ± 6	241 ± 4
Rural area-1	7.39 ± 0.30	2.02 ± 0.02	0.58 ± 0.03	3.62 ± 0.03	2.79 ± 0.02	2.14 ± 0.11	368 ± 21	22 ± 1	6 ± 0	30 ± 1	288 ± 17	49 ± 4
Rural area-2	8.25 ± 0.64	2.44 ± 0.13	0.48 ± 0.03	4.31 ± 0.25	1.97 ± 0.10	2.01 ± 0.14	258 ± 10	42 ± 14	8 ± 0	25 ± 2	216 ± 5	43 ± 1
Rural area-3	8.64 ± 0.06	2.25 ± 0.03	0.66 ± 0.01	4.03 ± 0.07	2.36 ± 0.07	2.68 ± 0.03	394 ± 9	40 ± 0	12 ± 0	43 ± 5	380 ± 18	61 ± 1

**Table 2:** Correlation matrix for the metal concentrations in street dust samples.

Elements	Al	Na	Mg	K	Ca	Mn	Cr	Fe	Ni	Cu	Zn
Na	−0.79 *										
Mg	−0.83 *	0.67									
K	0.77	−0.53	−0.94 *								
Ca	−0.76	0.82 *	0.77	−0.80							
Mn	−0.48	0.25	0.84 *	−0.90 *	0.54						
Cr	−0.90 *	0.93 *	0.89 *	−0.78	0.88 *	0.55					
Fe	−0.10	0.02	0.57	−0.70	0.46	0.84 *	0.28				
Ni	−0.90 *	0.94	0.87 *	−0.77	0.88 *	0.52	1.00 *	0.26			
Cu	−0.58	0.59	0.76	−0.82 *	0.91 *	0.66	0.74	0.73	0.74		
Zn	−0.21	0.18	0.48	−0.66	0.67	0.62	0.35	0.87 *	0.35	0.88 *	
Pb	−0.20	0.23	0.44	−0.61	0.71	0.54	0.36	0.81 *	0.36	0.88 *	0.99 *

* *p* < 0.01 correlation is significant.

**Table 3:** General characteristics and depression level of workers in the traditional marketplace.

Variables	Total (*n* = 163)	Fish Market (*n* = 38)	Fruits Market (*n* = 73)	Medicinal Herb Market (*n* = 52)	F or χ^2^	*p* Value
	Mean ± SD					
Age (years)	59.43 ± 9.35	60.58 ± 9.26 a	59.71 ± 8.22 a	59.31 ± 10.85 a	0.691	0.503
Working hours (hour/day)	11.44 ± 2.21	11.75 ± 2.05 a	11.35 ± 2.53 a	11.36 ± 1.81 a	0.471	0.625
Work experience (years)	21.61 ± 11.83	21.89 ± 9.36 a	21.14 ± 11.05 a	22.08 ± 14.32 a	0.109	0.897
Street venders (%)	34.4	28.6	43.8	14.3	12.22	0.002
Depression level (CES-D)	16.77 ± 8.78	21.13 ± 9.08 a	14.34 ± 8.56 b	16.98 ± 7.61 b	8.190	<0.001

Means with the same letter (a or b) are not significantly different at *p* < 0.005.
